# Microsurgical resection of a glioblastoma multiforme of the medulla oblongata with intraoperative subcortical stimulation and mapping

**DOI:** 10.3171/2019.10.FocusVid.19405

**Published:** 2019-10-01

**Authors:** Sima Sayyahmelli, Jian Ruan, Bryan Wheeler, Mustafa K. Başkaya

**Affiliations:** Department of Neurological Surgery, University of Wisconsin–Madison School of Medicine and Public Health, Madison, Wisconsin

**Keywords:** glioblastoma multiforme, infiltrative, lower cranial nerves, medulla oblongata, subcortical stimulation, video

## Abstract

Primary glioblastoma multiforme tumors of the medulla oblongata are rare, especially in the adult population. Perhaps due to this rarity, we are not aware of any previous reports addressing the resection of these tumors or their clinical outcomes.

In this surgical video, we present a 43-year-old man with a 1-month history of left-sided paresthesia. The paresthesia initiated in the left hand, along with weakness and reduced fine motor control, and then spread to the entire left side of the body. He had recent weight loss, imbalance, difficulty in swallowing, and hoarseness in his voice. He also had a diminished gag reflex, and significant atrophy of the right side of the tongue with an accompanying deviation of the uvula and fasciculations of the tongue. MRI showed an infiltrative expansile mass within the medulla with peripheral enhancement and central necrosis. In T2/FLAIR sequences, a hyperintense signal extended superiorly into the left inferior aspect of the pons and left inferior cerebellar peduncle and inferiorly into the upper cervical cord.

The decision was made to proceed with surgical resection. The patient underwent a midline suboccipital craniotomy with C1 laminectomy for surgical resection of this infiltrative expansile intrinsic mass in the medulla oblongata, with concurrent monitoring of motor and somatosensory evoked potentials and monitoring of lower cranial nerves IX, X, XI, and XII. A gross-total resection of the enhancing portion of the tumor was performed, along with a subtotal resection of the nonenhancing portion. The surgery and postoperative course were uneventful. Histopathology revealed a grade IV astrocytoma. The patient received radiation therapy.

In this surgical video, we demonstrate important steps for the microsurgical resection of this challenging glioblastoma multiforme of the medulla oblongata.

The video can be found here: https://youtu.be/QHbOVxdxbeU.

## Transcript

In this surgical video, we demonstrate important steps for the microsurgical resection of a large challenging glioblastoma multiforme in the medulla oblongata with intraoperative subcortical stimulation and mapping.

The patient is a 43-year-old man with a **(0:33)** 1-month history of left-sided paresthesias. The paresthesia initiated in the left hand, along with weakness and reduced fine motor control, and then spread to the entire left side of the body. He had recent weight loss, imbalance, difficulty in swallowing, and hoarseness in his voice. He had also had a **(0:52)** diminished gag reflex, and significant atrophy of the right side of the tongue with an accompanying deviation of the uvula and fasciculations of the tongue. MRI **(1:01)** showed an infiltrative expansile mass within the medulla with peripheral enhancement and central necrosis. In T2/FLAIR sequences, a hyperintense signal extended superiorly into the left inferior aspect of the pons and left inferior cerebellar peduncle and inferiorly into the upper cervical cord.

The possible differential diagnosis was a high-grade glioma or ependymoma. The decision was made **(1:25)** to proceed with surgical resection. The patient underwent a midline suboccipital craniotomy with C1 laminectomy for surgical resection of the infiltrative expansile intrinsic mass in the medulla oblongata, with concurrent monitoring of motor and somatosensory evoked potentials and monitoring of lower cranial nerves IX, X, XI, and XII.

The patient was placed in the prone position **(1:47)**. A midline skin incision was marked just above the inion extending down to the level of C4 spinous process. A pericranial graft above the inion was harvested for duraplasty at closure **(1:56)**. A T-shape fascial incision was done and a muscle cuff along the nuchal line was left for muscle closure at the end of case. Paravertebral muscle dissection was then performed in a subperiostal plane in the midline nuchal avascular line and retracted laterally.

A midline suboccipital craniotomy **(2:16)** was then performed by placing two burr holes on either side of the occipital keel. The posterior arch of the C1 was also removed. We then opened the dura in a Y fashion **(2:27)**. Baseline neuromonitoring responses **(2:30)** of both somatosensory and motor evoked potentials were obtained before and after positioning and were continuously monitored throughout the remainder of the surgery.

Under microscopic magnification and illumination, the arachnoid of the cisterna magna was opened sharply with a diamond knife and microscissors **(2:40)**. The posterior aspect of the medulla oblongata and upper cervical spinal cord were inspected, and an expansile mass in the left half of the medulla was noted. The location of this mass was also confirmed with intraoperative neuronavigation **(2:54)**.

In this section of the video, we observe the inferior aspect of the fourth ventricle **(3:02)**. The stimulator was then used to ensure that we avoid the lower cranial nerve nuclei as we made the cortical incision in the medulla. Multiple locations were probed with the stimulator **(3:07)**. On the left side of the medulla, stimulation was negative at up to 1 mA at multiple spots. On the right side and on the ventricle surface of the firm mass, there was firing from the right side of the tongue at low stimulation. With stimulation, there were also positive responses from the tongue and vocal cords at 0.9 to 1 mA, mostly on the right side **(3:25)**. In this section of the video, we can see the stria medullaris at the floor of the fourth ventricle **(3:42)**. At the left side and centered on the central necrosis, which was negative for stimulation, the surface pial vasculature was coagulated and a small vertical incision was done sharply with a diamond knife **(3:54)**. With a spreading motion of the bipolar, the tissue was dissected with a small portion resected and sent off for intraoperative pathology. The pathology was later reported as compatible with a high-grade glioma.

We continued our dissection using microsurgical techniques, including blunt spreading with the bipolar, suction aspiration, and use of an ultrasonic aspirator. This was done in a stepwise fashion until the central necrotic tissue had all been resected, upon which we reached a surrounding firmer tissue which had a contrast-enhancing rim. Intermittently, subcortical stimulation was done and whenever it was negative, the resection was continued. The extent of resection was guided with neuronavigation and the aforementioned periodic use of subcortical stimulation. The goal of resection was to safely remove the entire contrast-enhancing rim and some nonenhancing T2 flair areas. Cautiously, we continued using subcortical stimulation to stay away from motor fibers, which were pushed anteriorly.

We then addressed the firmer portion of the tumor that were to the right and superior. On the right side, subcortical stimulation was positive for the right lower extremity and right hand at 2 mA **(5:06)**. The resection was continued at negative stimulation spots **(5:14)** until we reached the part of the resection cavity which was positive for right lower extremity and right hand at 1.5 to 2 mA **(5:18)**. When we felt the resection was sufficient, with a margin of safety, we elected to stop. A final test of the motor and somatosensory evoked potentials was done and were unchanged **(5:30)**.

Hemostasis was obtained in the tumor cavity. The dura was then closed in a watertight fashion using the previously harvested pericranial tissue for grafting **(5:38)**. The craniectomy site was covered with titanium mesh **(5:45)**. The wound was then closed in layers.

The surgery and postoperative course were uneventful. Histopathology revealed a grade IV astrocytoma. The patient is currently about to complete radiation therapy.

In conclusion, in some selected high-grade glioma cases of the medulla oblongata, gross-total resection of the contrast-enhancing portions of the tumor can be accomplished. In our experience, gliomas of the medulla tend to occupy only one side of the brainstem, which may allow maximal safe resection.

Our aim is to achieve maximal resection without functional compromise with both low- and high-grade gliomas, since the mounting evidence suggests that more extensive surgical resection is associated with longer life expectancy in all high-grade gliomas. Although primary GBMs of the medulla oblongata are a rarity, and while initial reports documented no survival with or without treatment, recent reports have documented a longer median survival after tumor removal and radiation and chemotherapy.^4–6^

In the report by Yoshikawa et al.,^3^ radiation and chemotherapy was performed without biopsy and the patient died from respiratory arrest 18 days after administration of chemoradiotherapy. The autopsy revealed glioblastoma. Thus, better treatment options are desired. We believe that in our current era of microsurgery with advanced neuromonitoring adjuncts, in selected cases, gross-total resection can be accomplished. In our viewpoint, just performing a biopsy is not acceptable in such cases. A major objective of our video is to suggest that maximal safe resection with chemoradiation is a preferable option to improve the median survival of these challenging histopathology in the eloquent medulla oblongata, in contrast to traditional, no treatment, or conservative treatment.

Preoperative assessment of motor fibers with diffusion tractography MRI is limited in this tight part of the brainstem comparing to other parts of the brainstem. Although a thorough understanding of the anatomy of the brainstem safe entry zones is of great importance, electrophysiological mapping provides a safer approach to this part of the brainstem since tumors can alter the anatomy. In our opinion, although we are still in learning phase of the subcortical stimulation in the brainstem, this approach may be helpful to avoid causing iatrogenic motor deficits.
